# Widely Tunable Single/Dual RF Signal Generation by a Monolithic Three-Section DFB Laser

**DOI:** 10.1186/s11671-020-03317-w

**Published:** 2020-04-17

**Authors:** Chung-Ping Huang, Yu-Ming Huang, Hsiang-Yun Shih, Fu-Chun Hsiao, Chun-Hong Chen, Shun-Chieh Hsu, Chien-Chung Lin

**Affiliations:** 1 Institute of Lighting and Energy Photonics, College of Photonics, National Chiao-Tung University, Tainan, Taiwan; 2 Institute of Photonic System, College of Photonics, National Chiao-Tung University, Tainan, Taiwan; 3 Institute of Imaging and Biomedical Photonics, College of Photonics, National Chiao-Tung University, Tainan, Taiwan

**Keywords:** DFB lasers, FWM, Microwave generation, Monolithic integration

## Abstract

A three-section distributed feedback laser with a 2.5 InP/air pair of distributed Bragg reflectors (DBRs) was fabricated and analyzed in terms of its microwave generation capability. A widely tunable single radio frequency (RF) signal can be detected using optical heterodyning, and the tuning range is from 2 to 45 GHz. The incorporation of the third section provides an opportunity to present the dual RF operation when three emission peaks are near to each other in the wavelength domain. The proposed design provides a 21.3% enhancement in the RF tuning range compared with the range of a two-section laser (35.29 GHz versus 42.81 GHz). The compactness of the proposed device can be useful for future radio-over-fiber applications.

## Introduction

With the advent of future novel wireless technology, the installation of cellular network has evolved to a new era: a large number of micro- or nano-sized base stations are required, and a power-efficient microwave transmission can be expected [1, 2]. To construct this wireless infrastructure, a good microwave source is necessary. In the past, several methods have been proposed and demonstrated to generate microwaves (such as X/Ka-bands). Using electron-beam and backward oscillators (BWO) can provide high intensity of microwaves (usually in the range of several hundred megawatts to even giga-watts), and they are widely applied in the field of radar, remote sensing, communications, and plasma sciences [3–5]. However, this technology is difficult to tune the emission frequency because it is pre-determined by the fixed wave-guiding structure, and the size of this structure is usually in millimeter or centimeter. Another method is to apply transferred electron effect in a Gunn diode [6–9]. The semiconductor feature of the Gunn diode is very attractive as its size can range from tens of microns to even sub-microns. The delivered power output is also impressive: from several to tens of milliwatts. But the device usually requires other circuitry to provide good signal, and it also has a limited frequency tunability which is bounded by inherent carrier transmit time across the length of the device [10].

In addition to these traditional methods, the future wireless base station requires not only high efficiency but also small footprint and large-scale deployment. A small station architecture and the implementation of a massive multiple-input and multiple-output system demonstrate the need for microwave photonics [11]. Photonic devices and infrastructures can reduce the complexity of a network, increase transmission distance, and enhance transmission security. A combination of a picocell (small cell) and a fiber network can efficiently transmit a large quantity of data over a long distance [12]. Therefore, a different type of photonic device is necessary to realize such schemes, especially to generate a strong RF signal with high tunability and to enable multi-tasking. An injection-locked laser system was proposed for narrow linewidth RF generation [13]. Heterodyning multiple lasers with an optical phase-locked loop has been used to generate high-quality single- or dual-channel of RF signals and other circuitry to provide good signal, and data transmission can be shown in these schemes previously [14–16]. Multiple laser integration for microwave generation can be realized using arrayed waveguide grating (AWG) integration [17] and a serial cascaded programmable interrupt controller [18]. All these studies have relied on precisely aligned optics and multiple laser sources to provide sufficient photons to interact.

To further reduce the required footprint of the system, an integrated design is necessary. Considering all the methods that were previously published, we believe integrated microwave photonic generation could be a good candidate [18] because (a) the size of the chip can be scaled down similar to Si wafers. The current size of our photonic chips can range from tens to hundreds of microns, but further reduction in footprint is possible. (b) Photonic mixing can provide some of the best RF signals in the past literatures. For example, by using injection locking scheme, the phase noise can be greatly reduced, which is very important for the RF signal [19]. (c) External electrical current for widely tunable RF signal. By adjusting injection currents, the microwave photonic chips can easily realize a wide range of frequency generation via various interactions of photons such as refractive index change or optical heterodyne, etc. [20, 21]. The variety of physical properties of photons makes photonic chip very versatile in terms of frequency tuning. To fully utilize the aforementioned photonic advantages, different colors of coherent photons shall be able to be integrated in this chip design. In this study, a three-section distributed feedback (DFB) laser with DBR optical isolation was developed for the first time. The proposed laser can operate as either a simple tunable RF carrier or a carrier and data source with two RF tones. The characteristics of this integrated device can be fully investigated and analyzed, and we posit that this device can be beneficial for future microwave photonic integration.

## Methods

### Device Fabrication

**Fig. 1:**
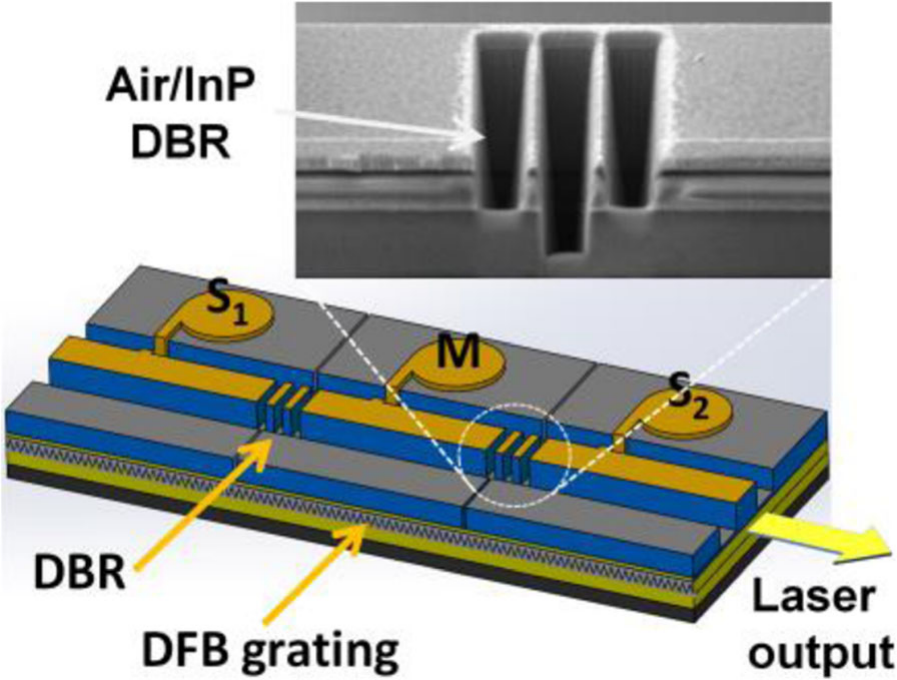
Schematic and SEM image of the three-section DFB laser device

In this study, wafers were first grown using a metalorganic chemical vapor deposition system. InGaAsP quantum wells were used as the active region, and the targeted lasing wavelength was approximately 1550 nm. The gratings of the DFB lasers were manufactured using e-beam lithography. After the epitaxial procedure was completed, the wafer was processed with the standard semiconductor processes of film deposition, dry/wet etching, and metallization that are described in [21]. The wafer was thinned down to 100 μm and polished for backside metal contact deposition (AuGe/Ni/Au) to finishing all the processing steps. The next step would be cutting the wafer into bars and dicing the bars into chips for packaging, and the size of the chip is 250 × 900 μm^2^. The integrated laser chip was attached on a ceramic submount and wire bonded for probing and testing. An air/semiconductor distributed Bragg reflector was etched using a nanoscale focused ion beam (FIB) system (Tescan model no. GAIA3). FIB technology employs accelerated Ga ions with 30 keV energy and 0.4 nA beam current to bombard the target semiconductor (such as InP or Si) away. With its nanometer scale accuracy, the FIB system can realize the inter-section DBR for the three-section laser. The DBR is composed of air and InP sections with a width of 1162 nm for the air section and 584 nm for InP section. The deepest etch is 7 μm into the wafer. To control interfacial roughness of air/semiconductor, we optimized the FIB etch rate to 33 nm/s. Figure 1 displays the schematic and SEM image of the finished device. The 2.5 pairs of air/InP DBRs between the section can provide both high optical reflectivity and electrical isolation, and they divide an integrated chip into three sections: S_1_, M, and S_2_, as shown in Fig. 1. We adapt the notation from injection locking lasers in which the master and slave lasers are commonly used for pumping and pumped devices.

### Optical Heterodyning

Optical heterodyning is a method of generating an RF signal in many microwave photonic structures [22, 23]. This technique generates a tunable RF signal by mixing different wavelengths of photons [24, 25]. First, we defined the two different signals *E*_1_ and *E*_2_ as follows:

1 $$ {E}_1={\mathrm{E}}_{01}\left({\upomega}_1t+{\upvarphi}_1\right), $$

2 $$ {E}_2={\mathrm{E}}_{02}\left({\upomega}_2t+{\upvarphi}_2\right), $$

where *E*_01_ and *E*_02_ are the amplitudes, *ω*_1_ and *ω*_2_ are the frequencies, and *φ*_1_ and *φ*_2_ are the phases corresponding to *E*_1_ and *E*_2_, respectively. Then, the intensity of the total mixing signal *I*_*t*_ can be described as follows [26]:

3 $$ {I}_t={\left({E}_1+{E}_2\right)}^2={E_{01}}^2{\mathit{\cos}}^2\left({\upomega}_1\mathrm{t}+{\upvarphi}_1\right)+{E_{02}}^2{\mathit{\cos}}^2\left({\upomega}_2\mathrm{t}+{\upvarphi}_2\right)+{E}_{01}{E}_{02}\left\{\mathit{\cos}\left[\left({\upomega}_1+{\upomega}_2\right)t+\left({\upvarphi}_1+{\upvarphi}_2\right)\right]+\mathit{\cos}\left[\left({\upomega}_1-{\upomega}_2\right)t+\left({\upvarphi}_1-{\upvarphi}_2\right)\right]\right\}, $$

While measuring the total signal, only the *E*_01_*E*_02_ × cos[(ω_1_ − ω_2_)*t* + (φ_1_ − φ_2_)] term can be observed because the high-frequency terms (such as *ω*_1_ and *ω*_2_ and *ω*_1_ + *ω*_2_) are above the detection limit of the photodetector. The final detected RF signal is obtained at the following frequency:

4 $$ \Delta \mathrm{f}=\mathrm{c}\;\left(\frac{1}{\lambda_1}\hbox{-} \frac{1}{\lambda_2}\right) $$

In the current device, photons with multiple wavelengths can be generated simultaneously such that a heterodyne can occur at different frequencies simultaneously. Because the emission wavelength of each laser can be controlled by the injection current, various combinations of currents can provide single and dual RF output signals from the same device. These conditions are discussed later in the paper.

### Measurement System

To appropriately evaluate laser devices, the output power is carefully fiber coupled into a calibrated photodetector (PD). The fiber end was cleaved at an 8° tilt to reduce the facet reflection. A polarization controller and proper isolators were installed to ensure minimum feedback to the laser and maximum output power after heterodyning. An erbium-doped fiber amplifier is an optional piece of equipment that can be neglected if the signal is sufficiently strong. A high-speed photodetector (a 50-GHz PD, u2tPhotonics®, AG) or another PD (1414, New Focus®) was used to detect the mixed photonic signal. The electrical signal obtained after heterodyning was input into a signal analyzer (N9030PXA, Keysight®), and the differential frequency spectrum of the signal was presented. On the other side, the combined optical spectrum was read using an optical spectrum analyzer (OSA; AQ6317B, Ando®).

## Results

### DC Device Characteristics

**Fig. 2:**
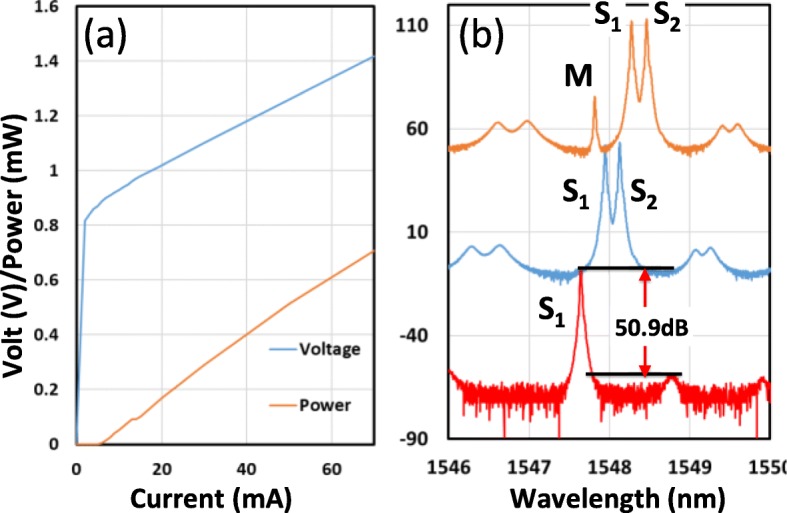
**a** Generic LIV curves of the DFB laser. **b** Optical spectrum with one, two, and three DFB lasers switched on

**Fig. 3:**
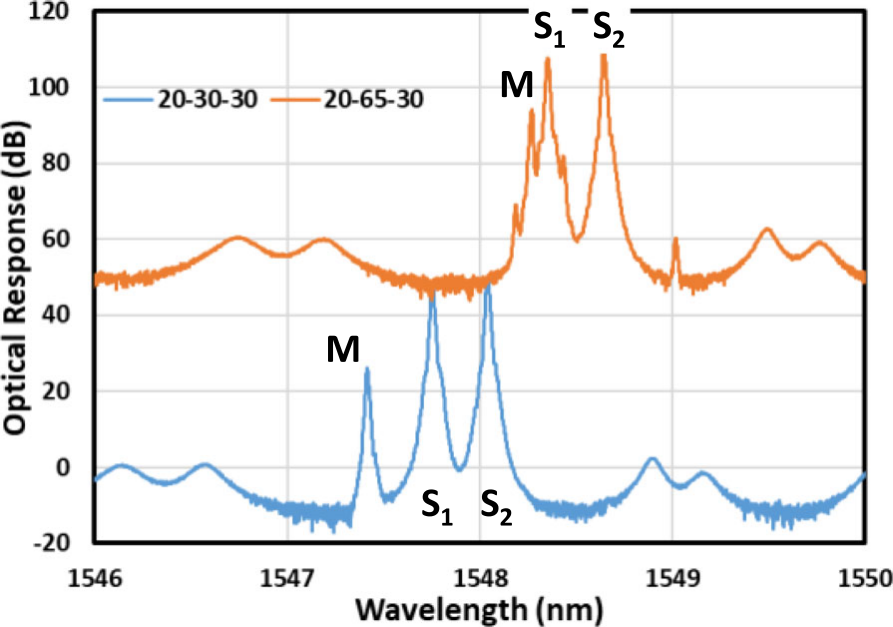
Optical spectrum of a three-section laser with and without the FWM effect. The legend presents the current combination (in mA) of S_1_–M–S_2_ section in each spectrum

Once device fabrication is completed, the DC characteristics can be tested. Figure 2a displays the generic power–current–voltage (L–I–V) curves of a generic DFB laser fabricated using this wafer. The threshold current can be smaller than 10 mA. The individual devices have a 300-μm long cavity and output power in the order of milliwatts. The grating in the structure provides necessary feedback and mode selection to allow the laser to operate in the single mode. The power spectrum observed when the three lasers are switched on is presented in Fig. 2b. A high side mode suppression ratio larger than 50 dB was measured for the single DFB case. Favorable single-mode operation is essential for the optical heterodyning to be successful. When the optical signals are taken from the two sides, the S_1_ and S_2_ sections exhibit a stronger response compared with the response of the middle section (M section), as presented in Fig. 2b because of the high reflection from the central DBR sections that block the output power from the M section. The optical mode spacing can be changed using the electrical injection currents. This flexibility provides a variety of combinations of the three modes of these lasers. Figure 3 displays the current-dependent optical spectra. All the three peaks can be adjusted, and the spacing between two peaks can be critical for the RF signal generation. When the two peaks are sufficiently close, four-wave mixing (FWM) occurs between these two wavelengths of photons [27]. When two peaks are far apart, no FWM effect is present. The FWM enhances from the nonlinear modulation of carrier concentration in the laser gain medium [27]. The modulation leads to the stronger heterodyne effect among different colors of photons and can produce a stronger RF output signal. In the top curve presented in Fig. 3, several peaks are generated in the optical spectrum due to this strong FWM interaction. The spacing between the peaks is still the same as the difference between the two original mixed frequencies.

### Widely Tunable Single-Mode RF Generation

**Fig. 4.**
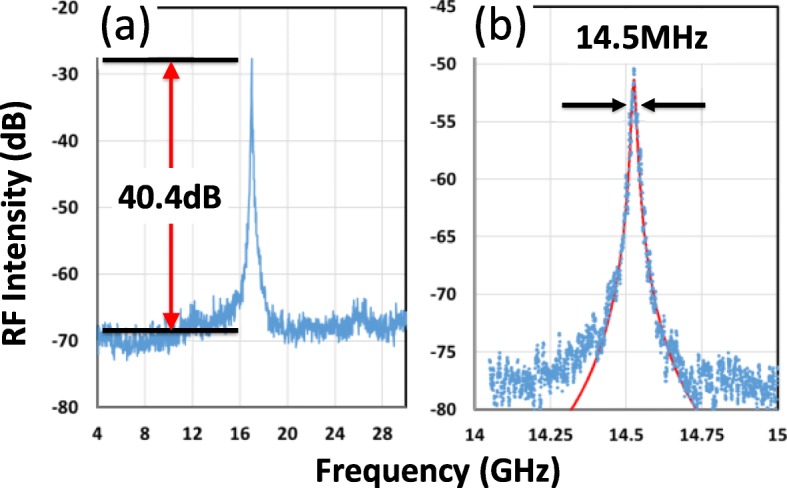
Electrical spectra of the synthesized RF signal. **a** Single mode RF signal. **b** The linewidth estimation of the single mode RF signal

When the injection current varies, the emission wavelength of the DFB laser varies, as aforementioned. Thus, the outcome of optical heterodyning changes accordingly in this device. The heterodyned RF signal can be measured using a high-speed photodetector [20]. The quality of the signal can be identified using a PXA setup. Figure 4a displays detailed electrical spectra of the synthesized RF signal. The single-mode signal rises 40.4 dB above the noise floor, and the peak intensity can be as high as − 20 dB. The finer resolution on the RF spectrum reveals the details of the signal, and the spectrum can be fitted using the Lorentzian function to determine the linewidth. The usual linewidth is approximately 12 to 16 MHz, as shown in Fig. 4b. The individual linewidth of the RF peak is defined by the addition of the linewidths of the peaks of the DFB lasers, which ranges from 5 to 7 MHz in this wafer. One of the important features of this design is the widely tunable single-mode RF generation. The combination of the three laser tones provides a wider RF distribution range. A single-mode RF signal can be continuously tuned from 2 to 45 GHz.

### Dual RF Mode Operation

**Fig. 5:**
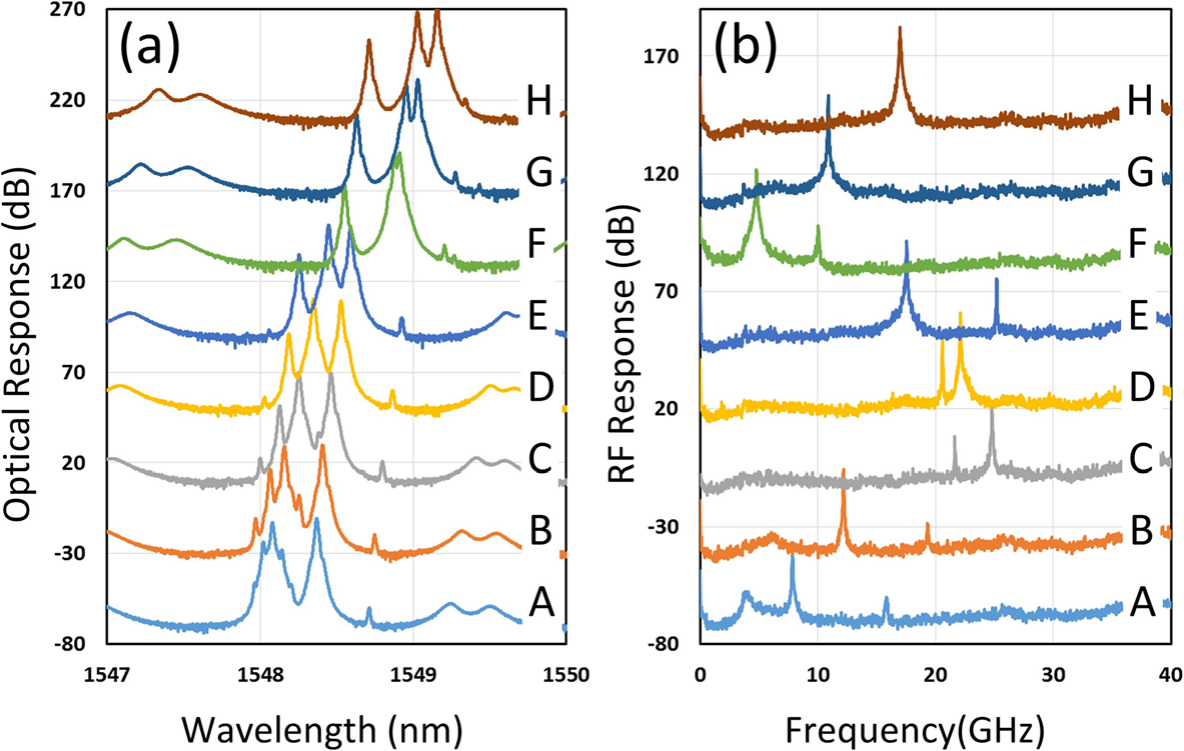
Dual-mode **a** optical and **b** RF spectra under different current combinations. The currents of the S_2_ and M sections are kept the same while the current of the S_1_ section is varied from 20 to 70 mA (shown in **a**). In the trace A, λ_M_<λ_S1_<λ_S2_, and the sequence becomes λ_M_<λ_S2_<λ_S1_ in the trace H

Due to the additional third section in the laser, the integrated device can provide more complicated RF signal patterns than the lasers with two sections. The dual RF mode in a controllable manner could be a favorable feature for various purposes. In this device, the dual mode occurs only when the three wavelengths of the lasers are close to each other. When the FWM effect can be initiated by all three lasers, two RF signals with different frequencies are observed. In Fig. 5, both optical and electrical spectra are displayed side by side to illustrate this scenario. In this figure, the peaks of sections S_1_ and M are near at the low current level. Thus, a strong FWM occurs between the S_1_ and M sections, and a strong RF peak is produced around 7.86 GHz (trace A). We increased the current of section S_1_ to red-shift its peak toward section S_2_. The major RF peak frequency increases when the separation between S_1_ and M sections becomes large (trace B). However, as the peaks of the S_1_ and S_2_ sections move closer, the heterodyning effect between these two groups of photons grows stronger. Thus, in trace C, the major RF signal becomes the differential frequency of S_1_ and S_2_. Moreover, the interaction between S_1_ and M remains, and a weaker RF signal corresponding to this interaction is observed at 21.6 GHz. By further increasing the current to S_1_, the major peak decreases in frequency because the peak of the section S_1_ red shifts toward the peak of the section S_2_. Meanwhile, the minor peak blue shifts to a higher frequency because the peak of section S_1_ moves away from the peak of section M (trace C to E).

In trace F, G, and H, the distance between the emission peak of section S_1_ and M is very large. Thus, no mixing occurs between these two sections, and S_1_ gradually surpasses S_2_ when the current of S_1_ is increased. The resulting RF peak first reduces and then increases in terms of frequency. This behavior is similar to the previously demonstrated two-section laser.

## Discussion

### Effect of the Number of Pairs

The DBR is inserted between the lasers to provide optical isolation between the cavities, to provide sufficient reflection between the two facets of each section of the DFB laser to increase the likelihood of obtaining single-mode emission, and finally, to provide sufficient electrical isolation between the sections. If the number of pairs is very small, then the electrical isolation might not be sufficient to maintain the independent pumping between the sections. Because the resistance of an individual laser diode is approximately equal to or less than 10 Ω, an electrical isolation of 10^3^ Ω or higher is preferred. Moreover, if the number of DBR pairs is very small, the individual sections cannot differentiate their own front or back facet reflectivities, and this can lead to unpredictable lasing modes in the front and back sections (S_1_ and S_2_). For the middle section (M section), fewer pairs of DBRs cause an inferior resonant condition and low cavity finesse, thus leading to no lasing at all. Conversely, if the number of DBR pairs is too large, the central section can lase in the multiple mode. Such lasing causes very little, sometimes zero, RF output.

### Function of the Middle Section

**Fig. 6:**
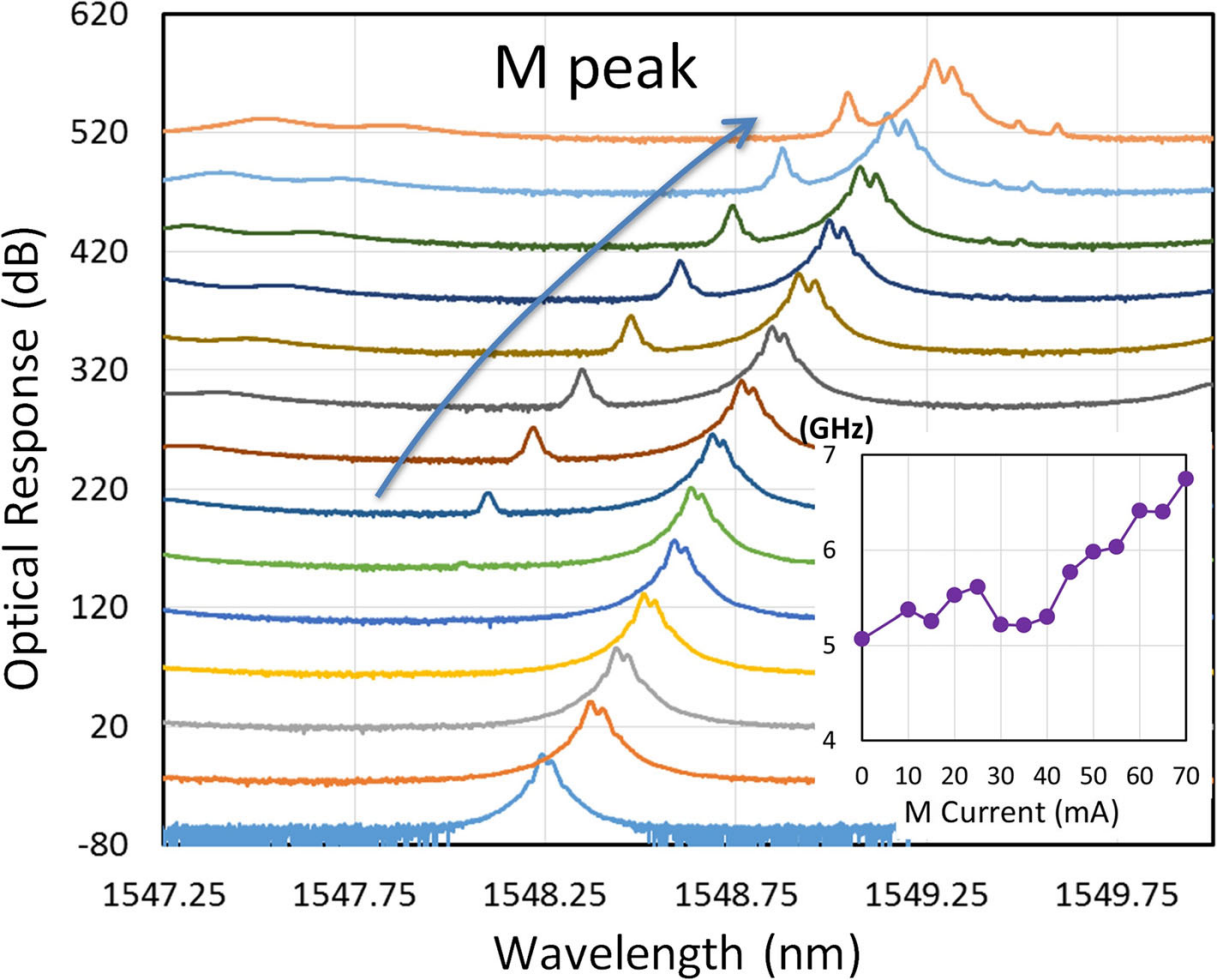
Optical spectrum of a three-section laser with two side sections (S_1_ and S_2_) with fixed inputs. The current injected to the middle section (M) increases from 0 to 70 mA. The inset presents the corresponding increase frequency in the RFs

Due to the limited FWM range in our two-section devices, the RF peak tuning was sometimes bound between 20 and 30 GHz. The strongly coupled two-section laser can also produce many complicated nonlinear modes of operation such as period 1 and chaos, as demonstrated previously [20]. When the third section was inserted into the laser chip, the tuning range was improved due to the extra thermal tuning effect of the devices. As shown in Fig. 6, when the currents of the S_1_ and S_2_ sections are fixed, the linearly varying current of the M section can provide an additional increase in RF tuning of 1.68 GHz. The obtained peak of the M section does not cause a strong optical mixing, and thus, all the major RF interactions are between the photons of the S_1_ and S_2_ sections. The slight increase in peak separation can also be observed in the traces with high input currents of the M section. In other devices, an increase in the RF of as high as 3.82 GHz was recorded. This additional change in synthesized RF frequency due to extra M section current can render the continuous tuning more feasible in the three-section laser. A comparison between *I*_M_ values of 0 and 65 mA in a specific device provides a difference of 7.52 GHz (Δ*f* = 42.81 GHz for *I*_M_ = 65 mA, and Δ*f* = 35.29 GHz for *I*_M_ = 0) in the tuning range. In general, several GHz tuning range can be added when M section is activated electrically and the change of current combinations can add up and increase the overall tuning range considerably. This phenomenon can be attributed to unequal thermal-wavelength dependence in the individual devices. The inequality is due to factors such as local doping variation, an uncut FIB region (causing leakage), and the nonlinear current dependence of the emission wavelength. In most cases, the RF peak frequency tends to increase when the third section is activated.

### Single or Dual Mode Operation

**Fig. 7:**
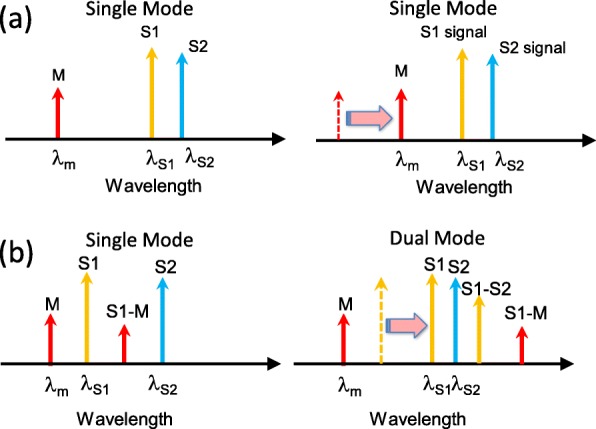
Comprehensive diagram of the operation modes for three-section DFB lasers: **a** One peak is far away and the other two are close to each other, and **b** all three peaks are close to each other

The analysis of the three-section laser operation appears complicated at first. In this paragraph, we bring perspective to a fundamental concern, whether the device will operate in the single or dual mode. Figure 7 presents the two most common modes of operation of our three-section laser. The mutual locations in the optical domain revealed that two cases were considered: in the first case, the third peak was far away from the remaining two peaks. In the second case, the third peak was actively close to the peaks in the S_1_ and S_2_ sections. In the first case, which is shown in Fig. 7a, the photons that are far away (peak of the M section) have very few interactions with the other two peaks (peaks of the S_1_ and S_2_ sections). Only the peaks of the S_1_ and S_2_ sections are sufficiently close to exhibit the FWM effect. In this condition, the three-section laser acts like the previously demonstrated two-section laser and a single RF peak is generated by mixing the peaks of the S_1_ and S_2_ sections. The function of the peak of the M section is to provide an extension or reduction of the RF peak based on the thermal-wavelength coefficients of the DFB sections. In the second case that is shown in Fig. 7b, the three peaks are near to each other. This case is more complicated. The proximity of photon wavelengths prompts the generation of the FWM effect, and more than one differential frequency can be generated due to this phenomenon. Thus, the top two combinations among the S_1_, S_2_, and M sections provide the constituent components in RF spectrum, and the laser can operate in the dual RF mode. However, once one of the FWM is weakened by the separation of the peaks due to current injection, the device returns to the single mode.

## Conclusions

A three-section laser was fabricated for the purpose of RF generation. In this laser, 2.5 InP/air pairs of DBRs were placed between the sections. This multiple section laser provides a single-mode RF signal that has high tunability from 2 to 45 GHz. The additional third section enables thermal tuning for this single-mode operation and also is essential for the dual RF mode operation. A strong FWM phenomenon was observed from the optical spectra and was confirmed by conducting RF peak measurement. The current-dependent wavelength-shift model can be applied for the verification of the RFs. The proposed three-section laser provides a 21.3% enhancement in the RF tuning range compared with the range of the two-section laser. In addition to the single-mode operation, a dual-mode RF signal was also demonstrated when the wavelengths of the three lasers are close to each other. The RF frequencies in the dual-mode operation can be modified by the direct current injection to any of the sections. We believe that the proposed laser will be useful for improving the performance of future microwave photonic devices and obtaining a highly efficient microwave photonic network.

## Data Availability

All the data and materials in the manuscript are available.

## Abbreviations

DBRs: Distributed Bragg reflectors
RF: Radiofrequency
AWG: Arrayed waveguide grating
BWO: Backward oscillators
DFB: Distributed feedback
FIB: Focused ion beam
PD: Photodetector
OSA: Optical spectrum analyzer
FWM: Four-wave mixing
